# Hepatic Presentation of Late-Onset Multiple Acyl-CoA Dehydrogenase Deficiency (MADD): Case Report and Systematic Review

**DOI:** 10.3389/fped.2021.672004

**Published:** 2021-05-10

**Authors:** Maria Anna Siano, Claudia Mandato, Lucia Nazzaro, Gennaro Iannicelli, Gian Paolo Ciccarelli, Ferdinando Barretta, Cristina Mazzaccara, Margherita Ruoppolo, Giulia Frisso, Carlo Baldi, Salvatore Tartaglione, Francesco Di Salle, Daniela Melis, Pietro Vajro

**Affiliations:** ^1^Postgraduate School of Pediatrics, Department of Medicine, Surgery and Dentistry “Scuola Medica Salernitana”, University of Salerno, Baronissi, Italy; ^2^Unit of Pediatrics 1, AORN Santobono-Pausilipon, Naples, Italy; ^3^Pediatric Clinic, AOU “S. Giovanni di Dio and Ruggi d'Aragona”, Salerno, Italy; ^4^Postgraduate School of Pediatrics, Faculty of Medicine University of Naples Federico II, Naples, Italy; ^5^Department of Molecular Medicine and Medical Biotechnology, Faculty of Medicine University of Naples Federico II, Naples, Italy; ^6^CEINGE-Biotecnologie Avanzate s.c.a r.l., Naples, Italy; ^7^Pathology Unit, AOU “S. Giovanni di Dio and Ruggi d'Aragona”, Salerno, Italy; ^8^Radiology Unit, AOU “S. Giovanni di Dio and Ruggi d'Aragona”, Salerno, Italy; ^9^Department of Medicine, Surgery and Dentistry “Scuola Medica Salernitana”, University of Salerno, Baronissi, Italy

**Keywords:** steatohepatitis, hypertransaminasemia, fatty liver, MADD, case report

## Abstract

Diagnosis of pediatric steatohepatitis is a challenging issue due to a vast number of established and novel causes. Here, we report a child with Multiple Acyl-CoA Dehydrogenase Deficiency (MADD) presenting with an underrated muscle weakness, exercise intolerance and an atypically severe steatotic liver involvement. A systematic literature review of liver involvement in MADD was performed as well. Our patient is a 11-year-old otherwise healthy, non-obese, male child admitted for some weakness/asthenia, vomiting and recurrent severe hypertransaminasemia (aspartate and alanine aminotransferases up to ×20 times upper limit of normal). Hepatic ultrasound showed a bright liver. MRI detected mild lipid storage of thighs muscles. A liver biopsy showed a micro-macrovacuolar steatohepatitis with minimal fibrosis. Main causes of hypertransaminasemia were ruled out. Serum aminoacids (increased proline), acylcarnitines (increased C4-C18) and a large excretion of urinary glutaric acid, ethylmalonic, butyric, isobutyric, 2-methyl-butyric and isovaleric acids suggested a diagnosis of MADD. Serum acylcarnitines and urinary organic acids fluctuated overtime paralleling serum transaminases during periods of illness/catabolic stress, confirming their recurrent nature. Genetic testing confirmed the diagnosis [homozygous c.1658A > G (p.Tyr553Cys) in exon 12 of the ETFDH gene]. Lipid-restricted diet and riboflavin treatment rapidly ameliorated symptoms, hepatic ultrasonography/enzymes, and metabolic profiles. Literature review (37 retrieved eligible studies, 283 patients) showed that liver is an extramuscular organ rarely involved in late-onset MADD (70 patients), and that amongst 45 patients who had fatty liver only nine had severe presentation.

**Conclusion:** MADD is a disorder with a clinically heterogeneous phenotype. Our study suggests that MADD warrants consideration in the work-up of obesity-unrelated severe steatohepatitis.

## Introduction

Multiple acyl-CoA dehydrogenase deficiency (MADD, MIM #231680), also known as Glutaric aciduria type II, is a rare autosomal recessive inherited disorder of fatty acid, amino acid, and choline metabolism. It is caused by deficiency of either an electron-transfer flavoprotein (ETF, encoded by ETFA and ETFB genes) or an electron-transfer flavoprotein dehydrogenase (ETFDH, encoded by ETFDH gene). The metabolic defects result in impaired adenosine triphosphate (ATP) biosynthesis, excessive lipid accumulation in different organs and insufficient gluconeogenesis (1).

The genetic heterogeneity correlates with different clinical phenotypes that can be divided into three types: (1) neonatal onset with congenital anomalies (MADD type I): the symptoms appear during the first 24 h of life and patients usually die within the first week of life; (2) neonatal onset without anomalies (MADD type II), with symptoms arising within the first 24–48 h of life and the death often occurs within the first weeks of life; (3) mild and/or late onset (MADD type III) with course and age at presentation extremely variable; in adolescents and adults muscular or cardiac symptoms are usually first features suggestive for MADD (2). Extramuscular symptoms such as fatty liver and recurrent vomiting have been reported more rarely (3). Patients with late-onset MADD carry at least one missense variation with minor amounts of residual ETF/ETFDH activity. This seems to be sufficient to prevent embryonic development of congenital anomalies. Instead, homozygosity for null mutations is usually associated with MADD type I (1).

Diagnosis is based on both the urinary organic acids profile and the blood acylcarnitine pattern. Urine presents elevations of glutaric, lactic, ethylmalonic, adipic, suberic, sebacic, butyric, and isovaleric acids, with elevation of 2-hydroxyglutaric acid being considered pathognomonic. The plasma acylcarnitine profile demonstrates generalized elevations in most of short, medium, and long chain acylcarnitines such as C4, C5, C5-DC, C6, C8, C10, C12, C14:1, C16, and C18:1. Biochemical diagnosis of patients with type III MADD is often very challenging, because elevation of urine organic acids may be incomplete, subtle, intermittent, or elevated only during an acute metabolic crisis (4).

Here, we report a 12-year-old male child presenting some muscle weakness and exercise intolerance along with an unusual extremely severe recurrent liver involvement, which has hitherto been rarely reported in type III MADD. A systematic literature review of liver involvement in MADD has been performed as well.

## Case Report

The proband was a 12-year-old otherwise healthy child admitted for some weakness/asthenia along with episodes of vomiting, and recently discovered severe hypertransaminasemia [alanine aminotransferase (ALT) and aspartate aminotransferase (AST) × 20 times upper limit of normal (uln)]. He was born at term after a normally conducted pregnancy. Previous regular checkup of laboratory tests including transaminases were normal at age 1, 7, and 8 years old. His early neurodevelopment was reported as normal with walking at 1 year of age and talking in phrases at 18 months of age.

He had a first access to a pediatric ward on July 2017 at age 11 for vomiting and asthenia. Laboratory showed hypertransaminasemia and increased serum values of creatine phosphokinase (CK) and lactic dehydrogenase (LDH) (ALT × 30 uln, AST × 20 uln, CK × 13 uln, and LDH × 6 uln), neutropenia (values at the entrance: WBC 3310/μL, N 650/μL) and positive serology of Parvovirus B19 infection [IgG 52,7 (positive if >11,5); IgM 98,5 (positive if >11,5)]. He was discharged after 10 days, following a sharp reduction of the values of serum enzymes and neutrophils normalization.

**Figure 1:**
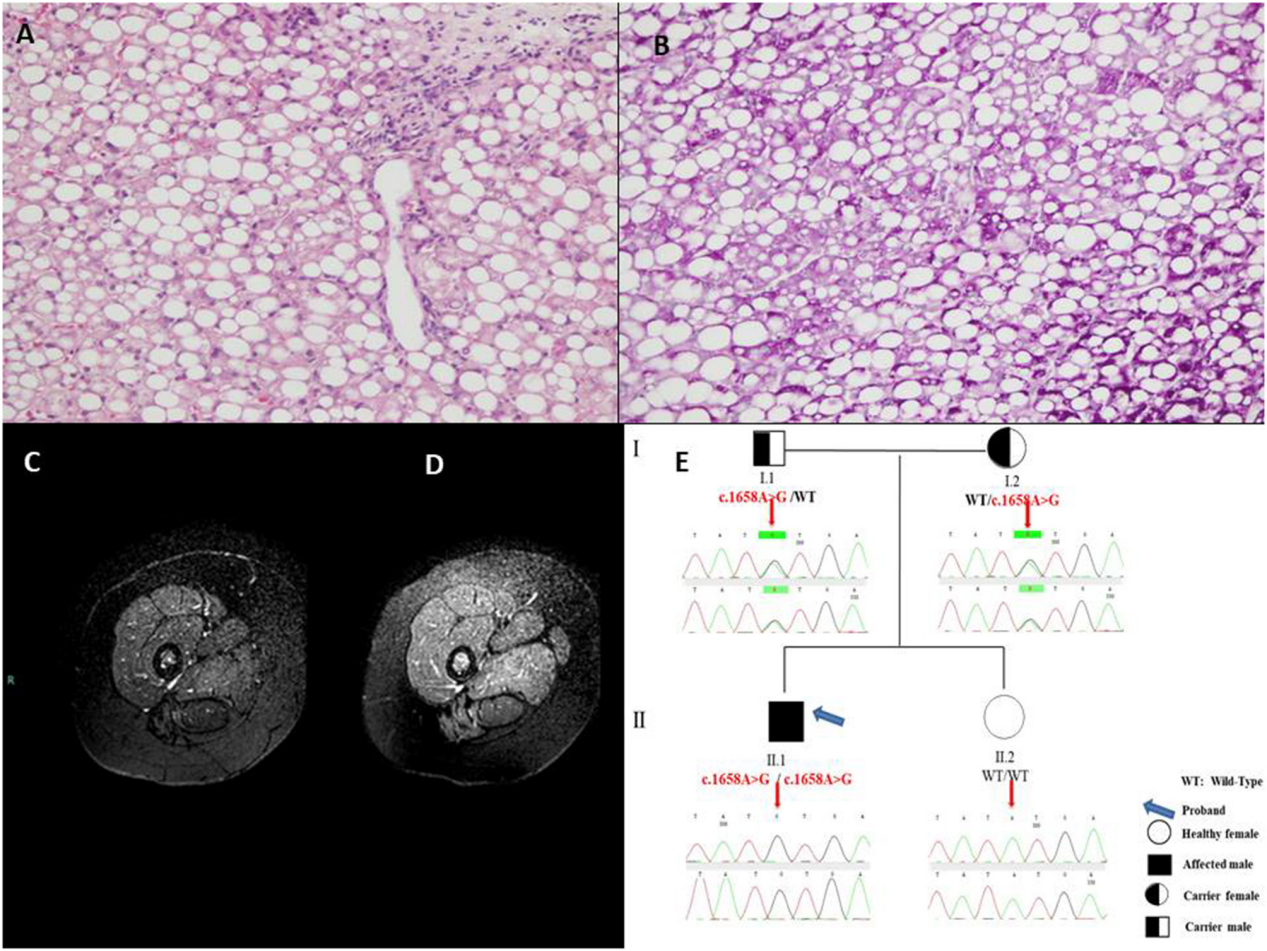
**(A)** Massive micro-macrovacuolar steatohepatitis picture with minimal fibrosis at liver biopsy with hematoxylin-eosin 10× and **(B)** Periodic Acid-Schiff, 20×. **(C)** Magnetic Resonance examination in the pre-treatment showed the overall signal from thigh muscles slightly reduced and clearly inhomogeneous, due to the hypointensity of posterior muscles, compatible with a diffuse fat infiltration in a Fat-Suppressed acquisition technique. **(D)** After 10-month treatment the overall Magnetic Resonance signal from thigh muscles is higher and less inhomogeneous, with a signal increase of the posterior muscles indicating a reduced fat infiltration. **(E)** Pedigree of the family of the patient with Multiple Acyl-CoA Dehydrogenase Deficiency (MADD).

In June 2019, he was admitted to our ward for a new episode of vomiting, asthenia, and hypertransaminasemia and neutropenia. His physical examination included a weight of 37 kg (50–75th percentile), height of 146.8 cm (50–75th percentile) and body mass index of 17.2 (50th percentile). There was no facial dysmorphism or significant skin finding. Muscle mass was normal with normal strength and tone. No tremor, ataxia, or abnormal movements were present. At entry laboratory data confirmed hypertransaminasemia (ALT × 30 uln; AST × 20 uln; CK × 10 uln; LDH × 4 uln). Hepatic ultrasound showed a severe bright liver. Cardiac sonography was normal. Due to a persistent elevation of serum enzymes, a liver biopsy was performed and it showed a massive micro-macrovacuolar steatohepatitis picture with minimal fibrosis without signs of inflammation (Figures 1A,B).

During the hospitalization, main viral [Hepatitis A, B, and C and minor hepatotropic viruses serology (e.g., Epstein Barr Virus and Cytomegalovirus)], autoimmune [anti - nuclear, smooth muscle, liver and kidney microsomes, liver cytosol, endomysium, tissue transglutaminase antibodies], toxic and common metabolic (Wilson disease, hereditary fructose intolerance) causes of hypertransaminasemia were ruled out by appropriate tests and anamnesis. Blood glucose, gases, lactate, ammonia were repeatedly found normal. Asthenia was evaluated by 6 min walking test which resulted positive showing fatigue after only 1.49 min (with oxygen saturation 78%, heart frequency 180 beats per minute, meters traveled 160). Magnetic Resonance Imaging [1.5 Tesla Philips MR equipment, Turbo Spin Echo Fat Suppressed T2w technique (TR 7014 ms, TE 100 ms, FA 90°), using 5 mm thick slices and 2 excitations] was indicative of lipid storage of thighs muscles (mild, Figure 1C), and confirmed liver steatosis (severe) as well. At hospital discharge after 11 days CK was normal (112 U/L), AST × 1.2 uln; ALT × 4 uln; LDH × 2 uln.

**Figure 2:**
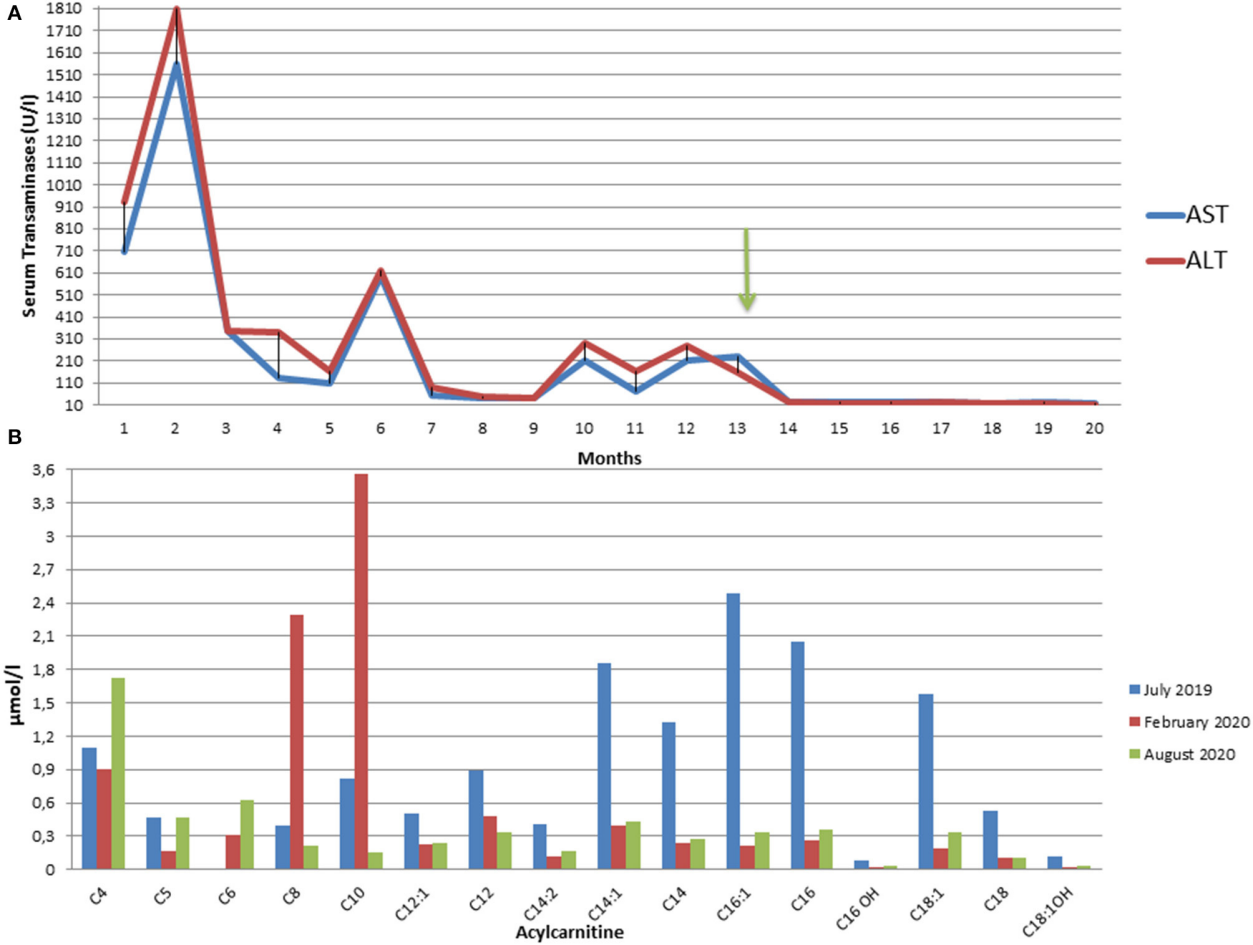
**(A)** Fluctuating trend of Aspartate (AST) and Alanine (ALT) aminotransferases from the onset of the symptoms to the present moment. The arrow indicates the start of riboflavin treatment. ALT and AST upper normal values are 40 U/L. **(B)** Plasma C3 to C16 acylcarnitine concentrations in our patient in July 2019 at the onset of symptoms (blue), in February 2020 after three months from the start of therapy (red), and in August 2020 after 9 months of therapy (green).

The determination of urinary organic acids and the search for serum amino acids and acyl carnitines were requested. Serum aminoacids showed increased proline; most of serum acylcarnites were increased (C4–C18) (Supplementary Table 1). Urinary mass spectrometric analysis showed a large excretion of glutaric acid, ethylmalonic, butyric, isobutyric, 2-methyl-butyric, and isovaleric acids suggesting a possible diagnosis of MADD. During follow-up, amounts of serum acylcarnitines and urinary organic acids fluctuated paralleling serum transaminases, with highest values manifest during periods of illness (e.g., flu) or catabolic stress (e.g., protracted fasting), confirming the recurrent nature of episodes in MADD (Supplementary Table 1 and Figures 2A,B).

After informed consent from the parents, a genetic test to confirm the diagnosis and to identify the carrier subjects in the family was therefore requested. Molecular analysis of genomic DNA isolated from peripheral whole blood was performed by Sanger sequencing of coding regions, intron/exon boundaries and 5′ and 3′ UTR of *ETFA, ETFB*, and *ETFDH* genes, using previously described protocol (5). Genetic results revealed the presence of the novel missense variant c.1658A > G (p.Tyr553Cys) in exon 12 of the *ETFDH* gene in the patient, at homozygous state. Both parents were heterozygous for the mutated allele. The proband's sister did not show the variant (Figure 1E).

Lipid restricted diet and Riboflavin treatment (100 mg thrice/day) ameliorated dramatically the asthenic symptoms. Repeat 6 min walking test showed no/minimal fatigue after 2 months (oxygen saturation 100%, heart frequency 150 beats per minute, 560 m traveled). Liver brightness at ultrasonography, liver function tests, along with the whole hepatic, muscular and metabolic laboratory profiles improved remarkably and persistently (Supplementary Table 1). Control Magnetic Resonance Imaging, after 10 months of therapy, showed absent/sharp decrease of lipid storage of thighs muscles (Figure 1D).

## Literature Review

We searched within the PubMed, Scopus, and Cochrane Library academic medical databases. The database search strategy was formulated around terms for MADD AND several other text words reported in Supplementary Table 2. Text words were chosen based on the existing literature and were obtained from related bibliographies. The earliest publication chosen date was January 2000 and the search ended in February 2021. Systematic search of literature was performed with no language restrictions. To be eligible for inclusion, studies had to describe a case of MADD associated with liver disease.

Study details and quality characteristics were independently extracted by three of the authors for all the articles and in a stepwise approach, first by reading the title, then by reviewing the abstract, and finally by revising the full text, where appropriate (Supplementary Figure 1). At the end of revision, 37 studies were selected.

Out of 270 reported patients, 70 had liver involvement. Fatty liver (45 cases) and recurrent vomiting represents rare extra-muscular symptoms of late onset MADD, and it has been describsed especially in mainland Chinese patients (3). Table 1 shows literature cases reported with fatty liver and/or liver disease as compared with our patient. The ages of the patients included ranged from six months to 68 years at diagnosis. Only 9 out of the 45 patients had severe fatty liver either at imaging or at liver histology. One patient showed early stages of liver failure, one recurrent pancreatitis, and one recurrent rhabdomyolysis and acute renal failure after the age of 46.

**Table 1:** Summary of the systematic review of the literature with studies reporting liver involvement in patients with Multiple Acyl-CoA Dehydrogenase Deficiency (MADD).

**Reference**	**Cases with hepatopathy /Total**	**Age onset Gender**	**ETFDH gene mutations**	**Main symptoms**	**Liver involvement**	**Reported Laboratory anomalies**
Al-Essa et al. (6)	1/7	7 y M	n.r.	Two to three metabolic crises per year, after meals rich in fat or protein.	US enlarged liver with normal echogenic pattern.	CK and hepatic profile (serum transaminases): normal
Liang et al. (7)	1	22 y F	n.r.	Recurrent pancreatitis; exercise intolerance, generalized muscle weakness with difficult walking, swallowing, and controlling the head. Four limbs areflexia.	US fatty liver, hepatomegaly, and enlarged pancreas with calcification. Liver biopsy: marked fatty metamorphosis, cytoplasm with prominent cell borders (plantlike appearance).	↑ CK ↑ AST, ALT ↑ 2-hydroxylglutaric, pyruvic, ethylmalonic, hippuric, adipic, and suberic acids. ↑ acylcarnitines
Gempel et al. (8)	1/7	34 yF	c.1367 C > T; c.1768 A > G	Insulin-dependent diabetes mellitus diagnosed at age 14. Proximal muscle weakness, exercise intolerance, hepatopathy.	Hepatopathy	↑ CK ↑ AST, ALT ↑ acylcarnitines
Yotsumoto et al. (9)	3/11	1 y 10 m, 5, 13 y3F	(1) 1096C > T; 1675C > T (2) c.1096C > T (3) c.524G > A; 1774T > C	(1) Vomiting, hypoglycemia, and liver dysfunction.(2) Convulsion, hypoglycemia, and liver dysfunction.(3) Vomiting, hypotonia, and liver dysfunction.	n.r	n.r.
Liang et al. (10)	1/4	10 yF	c.250G > A; c.380T > A	Mild weakness	Lipid storage disease at liver biopsy	↑ CK ↑ AST, ALT ↑ 2-hydroxylglutaric, ethylmalonic, glutaric and suberic acids. ↑ acylcarnitines
Ohkuma et al. (11)	2/4	5, 6 m1M, 1F	(1) c.1519T > G (2) c.1208C > T	Muscle weakness, hepatomegaly	Hepatomegaly	n.r.
Wen et al. (12)	1/17	15 yM	n.r.	Muscle weakness, muscle pain	US Moderate Fatty liver	↑ CK ↑ urinary organic acids ↑ acylcarnitines
Lan et al. (13)	3/9	7, 20, 18 y2M,1F	(1) c.250G > A (2) c.250G > A c.524G > A (+ PNPLA2 c.863C > G) (3) n.r.	(1) Encephalopathy, liver function impairment, lactic acidosis. Dysphagia. Myalgia and limb weakness.(2) Vomiting, liver function impairment. Myalgia and limb weakness.(3) Myalgia	Pt 3. Fatty liver	↑ CK ↑ acylcarnitines
Tojo et al. (14)	1	6 monthM	n.r.	Severe muscle weakness (lipid storage myopathy).	Fatty liver	↑ CK ↑ urinary organic acids
Wolfe et al. (15)	1	22 y M	c.820 G > T (exon 7) c.1601 C > T (exon 12)	Severe hypoglycemia and lethargy during an acute illness at 4 months of age. At age 4 years: recurrent vomiting, mild hyperammonemia and dehydration requiring frequent hospitalizations; generalized weakness associated with acute illnesses and growth spurts. Exercise intolerance and proximal muscle weakness.	Approximately 30% of the liver specimen demonstrated mixed macromicro-steatosis (biopsy).	↑ CK ↑ urinary organic acids
Xi et al. (16)	10/35	25 ± 13.6 y 12 F/18 M	n.r.	Muscle weakness and wasting, dysphagia, palpitation, vomiting.	US Fatty liver	n.r.
Izumi et al. (17)	1	54 y M	c.1211T > C	Recurrent rhabdomyolysis and acute renal failure after the age of 46; fatigability and weakness of neck and lower limbs.	US Fatty liver	↑ CK ↑ urinary organic acids ↑ acylcarnitines
Lammer et al. (18)	1	41y M	n.r.	General weakness	n.r.	↑ AST, ALT
Scheicht et al. (19)	1	22 y M	n.r.	Progressive reduction of physical overall performance, muscle weakness of the extremities, diarrhea.	Early stages of liver failure	↑ AST, ALT ↑ CK
Fitzgerald et al. (20)	1	22 y F	c.1367C > T	Cyclic vomiting syndrome diagnosed at 9 years and chronic fatigue. At 22 years, cardiovascular collapse, raised anion gap metabolic acidosis and non-ketotic hypoglycaemia. Post-mortem diagnosis of MADD (lipid storage involving the skeletal muscle, heart muscle, liver and cerebellum).	US and CT fatty infiltration of the liver with ascites	n.r.
Zhu et al. (3)	4/13	26, 32, 36, 38 y4M	(1) c.518T > G; c.1211T > C (2) c.1399G > A (3) c.715G > A; c.1810G > T (4) c.295C > T; c.821G > A	Muscle weakness, soreness, vomiting	US Severe Fatty liver	↑ CK
Xi et al. (21)	9/90	n.r.	n.r.	Muscle weakness, exercise intolerance, vomiting or diarrhea, palpitation.	US Fatty liver	n.r.
Rosenbohm et al. (22)	1	25 y F	c.1544G > T	Mild fluctuating weakness	US steatohepatitis, and hepatomegaly	↑ AST, ALT ↑ CK ↑ urinary organic acids ↑ acylcarnitines
Zhuo et al. (23)	1	9 y F	c.389A > T; c.736G > A	Muscle weakness, soreness, vomiting	Hepatomegaly	↑ AST, ALT ↑ CK ↑ urinary organic acids ↑ acylcarnitines
Ersoy et al. (24)	1	19 y F	n.r.	Progressive muscle weakness; intensive care unit with respiratory failure and acute renal failure.	Hepatomegaly	↑ AST, ALT ↑ CK ↑ acylcarnitines
Fu et al. (25)	2/3	23, 68 M	(1) c.250G > A; c.892C > T	(1) Muscle weakness; intermittent nausea and vomiting in the morning. Subacute premature fatigue, exercise intolerance, and weakness of shoulder, hip, neck, and masticatory muscles, general malaise and myalgia resembling polymyositis.	US Fatty liver	↑ AST, ALT ↑ CK ↑ urinary organic acids ↑ acylcarnitines
			(2) c.250G > A; c.449-453delTAACA	(2) Chronic or recurrent abdominal pains. Proximal limb and truncal muscle weakness, fatigue, myalgia, dysphagia, exertional and nocturnal dyspnea, and choking.		
Dai et al. (26)	1	13.83 y F	c.250G > A; c.353G > T	Muscle weakness	US Severe Fatty liver	↑ myocardial enzymes ↑ total cholesterol ↑ lactic acid ↑ serum free fatty acids
Yamada et al. (27)	2	58/31 y M	n.r.	Myalgia, muscle weakness	Liver dysfunction	
Behin et al. (28)	2/13	18, 22 y M/F	(1) c.1366C > T; c.1366C > T (2) c.877C > G; c.1691-3C > G	(1) “Transient hepatitis” with vomiting, abdominal pain, fatigue and myalgia during pregnancy. Exercise intolerance.(2) Delirium, vomiting, asthenia, exercise intolerance, progressive severe proximal and axial, weakness, bulbar signs.	Pt 2. Steatosis (liver biopsy)	↑ CK; ↑ C8 toC16; ↑ 2hydroxyglutaric acid, no acylglycines ↑ C4 to C18 ↑ Ethylmalonicacid
Cui et al. (29)	1	11 y M	n.r.	Muscle weakness, unconsciousness	US Fatty liver Hepatomegaly	↑ AST, ALT ↓ glucose Metabolic acidosis
Angelini et al. (30)	1/6	23 y F	c.451A > G exon 4 c.1649T > G exon 12	Progressive lower limb weakness and myalgias for 4 years; exercise intolerance, intermittent hypoglycaema.	US slight hepatomegaly	↑ CK
Missaglia et al. (2)	1	54 y F	c.1285 + 1G > A; c.560C > T	Recurrent episodes of vomiting, drowsiness, appetite loss, asthenia, and acetonemic breath; progressive arm, lower limb and neck flexors weakness.	Mild swelling of hepatocytes (liver biopsy)	↑ CK
Van der Westhuizen et al. (31)	2/3	2 y F 4 y F	(1) c.1067G > A; c.1448C > T (2) c.1067G > A; c.1448C > T	(1) Progressive muscle weakness. Hepatomegaly. Migraine. Exercise intolerance. Hypermobility(2) Progressive muscle weakness. Hepatomegaly. Migraine	Pt 2. macrovacuolar steatosis	↑ AST, ↑ ALT ↑ urinary organic acids ↑ acylcarnitines ↑ amino acids
Fan et al. (32)	2/4	30, 27 y 2M	(1) c.524G > A; c.1450T > C (2) c.1157G > A; c.1450T > C	(1) Muscle weakness(2) Non-ketotic hypoglycemia, metabolic acidosis, electrolyte disturbances, and increased muscle enzymes.	Pt 1. US fatty liver Pt 2. severe liver injury	↑ CK, ↑ AST ↑ urinary organic acids ↑ acylcarnitines
Chen et al. (33)	2 (Brothers)	19/13 y M	c.250 G > A	Exercise-induced myalgia, progressive proximal muscle weakness.	n.r.	↑ AST, ALT ↑ CK
Hu et al. (34)	1	6 m F	c.1812delG; c.579A > G	Muscle weakness. Crying faintly, vomiting and appetite loss.	TC scan fatty liver	↑ AST, ALT ↑ CK
Soldath et al. (35)	1	22 y M	c.1074G > C	Mild exercise intolerance; muscle weakness and pain (lipid storage myopathy). Crises of hypoglycemia and metabolic decompensation. Recurrent Clostridium difficile infection and septicemia.	Initially steatosis, then cirrhosis and acute liver failure.	↑ AST, ALT ↑ CK ↑↑ acyl-carnitines of all lengths
Santananukarn et al. (36)	1	40 y F	c.[250G > A]; [524G > A]	Subacute severe weakness of bulbar and limb muscles.	US Fatty liver Liver biopsy: severe steatosis	↑ AST, ALT ↑ CK
Pan et al. (37)	1	17 y F	c.250G > A; c.872T > G	Exercise intolerance and muscle weakness; palpitation and shortness of breath after exercise. Transient loss of consciousness. Muscle biopsy: lipid storage.	US Fatty liver	↑ AST, ALT ↑ acyl-carnitines
Xiao et al. (38, 39)	1	23 y M	c.665A > C; c.964G > T	Progressive exercise intolerance accompanied by predominantly lower proximal muscle weakness and pain.	US Fatty liver	↑ AST, ALT, ↑CK ↑ urinary organic acids ↑ acylcarnitines
Nilipour et al. (40)	2/29	3, 35 y 2F	c.1130T > C; c.92C > T	Muscle weakness	Hepatomegaly	↑ CK
Lian et al. (41)	1	n.r.	c.807A > C	Recurrent abdominal pain, vomiting, and impaired consciousness.	Fatty liver	n.r.
Present case	1	11 y M	c.1658 A > G	Vomiting, asthenia	US, CT scan and histologic fatty liver (severe)	↑ AST, ALT ↑ urinary organic acids ↑ acylcarnitines ↑ amino acids
TOTAL	70/283				46 fatty liver, of which 10 severe	

*↑ Increased. ETFDH, Electron-transfer flavoprotein dehydrogenase; ALT, Alanine amino transferases; AST, Aspartate amino transferases; CK, Creatine kinase; m, Month; Y, Year; M, Male; F, Female; US, Ultrasound; CT, Computerized tomography; n.r., Not reported*.

## Discussion

Differently from adult age, where the causes of hypertransaminasemia and/or fatty liver other than obesity-related liver disease are limited, the pediatric diagnostic approach may be more complex. In addition to nonalcoholic fatty liver disease (NAFLD)/metabolic dysfunction-associated fatty liver disease (MAFLD) children, in fact, may present individually rare genetic/metabolic conditions which collectively end up into a relevant and challenging ≥30% group of heterogeneous pediatric onset liver diseases (42–44). These, therefore, still require a cautious exclusion, so that pediatric fatty liver disease (PeFLD) with some subtypes has been proposed to be a better terminology to use and to be subject to revisitation upon reaching adulthood (45). In our patient urinary organic acids, serum amino acids and acyl carnitine patterns were crucial to suggest the diagnosis and pave the way to the subsequent confirmatory tests of a late onset MADD-related fatty liver disease. The latter is a condition which is more frequently characterized by prevalent metabolic and muscular signs rather than by severe fatty liver as confirmed by our systematic review of the literature (Table 1).

Molecular analysis of *ETFA, ETFB*, and *ETFDH* genes indicated that our patient was homozygous for the missense variant c.1658 A>G (p.Tyr553Cys) in exon 12 of the *ETFDH* gene. This variant is absent in the public database of mutations and polymorphisms. Interestingly, in the literature a pathogenic missense mutation (c.1657T > C) is reported in the same codon, determining a different amino acid substitution (p.Tyr553His). Including this observation in the pathogenetic evaluation, performed according to the American College of Medical Genetics and Genomics (ACMG) guidelines for variant interpretation (38), the novel variant c.1657T > C (p.Tyr553Cys) is classifiable as likely pathogenetic. Therefore, we identified a novel missense mutation in the *ETFDH* gene associated to late-onset riboflavin-responsive MADD.

The clinical phenotype of type III MADD is highly variable and ranges from acute, in some cases even fatal, metabolic crises in infancy to asymptomatic adults. This intriguing variability may probably be explained by the observation that even minute amounts of residual ETF/ETFDH activity seem to be sufficient to prevent embryonic development of congenital anomalies. Studies of an Asp128Asn mutation of the *ETFB* gene, identified in a patient with type III disease, showed that the residual activity of the enzyme could be rescued up to 59% of that of wild type activity when ETFB-(Asp128Asn)-transformed E. coli cells were grown at low temperature. This suggests that the environmental factors such as cellular temperature and stress may influence the enzymatic phenotype (46) in agreement with the case history of our patient. Although most patients become symptomatic within the first two decades, onset of symptoms ranges from the second month of life to late adulthood. Decompensations are characterized by acidosis, hypoglycemia, elevated activities of transaminases, rhabdomyolysis with raised creatine kinase activity, and, eventually, hyperammonemia. These episodes are usually triggered by catabolic states, either due to infections and febrile illnesses or to a reduced energy supply (1). In our case, the clinical presentation was prevalently characterized by some vomiting episodes and fatigue and recurrent severe hypertransaminasemia with a histological correlate of massive micro-macrovacuolar steatohepatitis with minimal fibrosis. Lipid restricted diet and riboflavin treatment dramatically ameliorated the clinical symptoms, liver brightness and LFTs, and metabolic profiles as reported in literature for the late onset MADD, which is therefore also called *riboflavin-responsive MADD* (RR-MADD) (3, 47). Riboflavin, the precursor of the coenzyme FAD, acts as a molecular chaperone that promotes folding and steady state levels of misfolded ETF-QO proteins in early stages and stabilizes folding intermediates or membrane-inserted proteins in later stages (48).

## Conclusions

RR-MADD is a treatable but rare disease and its diagnosis is difficult due to its high clinical phenotypic heterogeneity (13). Prevalent hepatic disease rather than isolated lipidic myopathy is a much less common presentation of the late-onset form. Our study is significant as it suggests that one should have consideration for this condition in the work-up of an otherwise orphan diagnosis of obesity-unrelated severe steatohepatitis with recurrent hypertransaminasemia. Correct diagnosis of MADD type III may allow timely initiation of a sometimes lifesaving riboflavin treatment and improve outcome.

## Data Availability Statement

The original contributions presented in the study are included in the article/Supplementary Material, further inquiries can be directed to the corresponding author/s.

## Ethics Statement

Ethical review and approval was not required for the study on human participants in accordance with the local legislation and institutional requirements. Written informed consent to participate in this study was provided by the participants' legal guardian/next of kin. Written informed consent was obtained from the minor(s)' legal guardian/next of kin for the publication of any potentially identifiable images or data included in this article.

## Author Contributions

MAS and CM conceived the original idea and took the lead in writing the manuscript with input from all authors. LN, GI, and GPC followed the clinical course of the patient over time. CB made the histopathology study. ST and FDS made MRI studies. FB, CM, MR, and GF carried out biochemical and molecular studies. DM and PV gave substantial intellectual contribution. PV is the guarantor of the study. All authors provided critical feedback and helped shape the study, discussed the results, and contributed to the final manuscript.

## Conflict of Interest

The authors declare that the research was conducted in the absence of any commercial or financial relationships that could be construed as a potential conflict of interest.

## Supplementary Material

Supplementary Figure 1 Flowchart of literature search results. Preferred Reporting Items for Systematic Reviews and Meta-Analyses (PRISMA) flowchart of literature search results. From: Moher D, Liberati A, Tetzlaff J, Altman DG, The PRISMA Group (2009). Preferred Reporting Items for Systematic Reviews and Meta-Analyses: The PRISMA Statement. PLoS Med 6(7):e1000097.

Supplementary Table 1 Serum acylcarnitines, aminoacids, and urinary organic acids at presentation and during the follow-up.

Supplementary Table 2 Key words used for PUBMED, Scopus and Cochrane library search.

## References

[1] Grünert SC. Clinical and genetical heterogeneity of late-onset multiple acyl-coenzyme A dehydrogenase deficiency. Orphanet J Rare Dis. (2014) 9:117. 10.1186/s13023-014-0117-5 25200064 PMC4222585

[2] Missaglia S Tavian D Moro L Angelini C. Characterization of two ETFDH mutations in a novel case of riboflavin-responsive multiple acyl-CoA dehydrogenase deficiency. Lipids Health Dis. (2018) 17:254. 10.1186/s12944-018-0903-5 30424791 PMC6234560

[3] Zhu M Zhu X Qi X Weijiang D Yu Y Wan H. Riboflavin-responsive multiple Acyl-CoA dehydrogenation deficiency in 13 cases, and a literature review in mainland Chinese patients. J Hum Genet. (2014) 59:256–61. 10.1038/jhg.2014.10 24522293

[4] Pollard LM Williams NR Espinoza L Wood TC Spector EB Schroer RJ. Diagnosis, treatment, and long-term outcomes of late-onset (type III) multiple acyl-CoA dehydrogenase deficiency. J Child Neurol. (2010) 25:954–60. 10.1177/0883073809351984 20023066

[5] Creanza A Cotugno M Mazzaccara C Frisso G Parenti G Capaldo B. Successful pregnancy in a young woman with multiple Acyl-CoA dehydrogenase deficiency. JIMD Rep. (2018) 39:1–6. 10.1007/8904_2017_38 28685490 PMC5953900

[6] Al-Essa MA Rashed MS Bakheet SM Patay ZJ Ozand PT. Glutaric aciduria type II: observations in seven patients with neonatal- and late-onset disease. J Perinatol. (2000) 20:120–8. 10.1038/sj.jp.7200325 10785889

[7] Liang WC Tsai KB Lai CL Chen LH Jong YJ. Riboflavin-responsive glutaric aciduria type II with recurrent pancreatitis. Pediatr Neurol. (2004) 31:218–21. 10.1016/j.pediatrneurol.2004.02.015 15351024

[8] Gempel K Topaloglu H Talim B Schneiderat P Schoser BG Hans VH. The myopathic form of coenzyme Q10 deficiency is caused by mutations in the electron-transferring-flavoprotein dehydrogenase (ETFDH) gene. Brain. (2007) 130:2037–44. 10.1093/brain/awm054 17412732 PMC4345103

[9] Yotsumoto Y Hasegawa Y Fukuda S Kobayashi H Endo M Fukao T. Clinical and molecular investigations of Japanese cases of glutaric acidemia type 2. Mol Genet Metab. (2008) 94:61–7. 10.1016/j.ymgme.2008.01.002 18289905

[10] Liang WC Ohkuma A Hayashi YK López LC Hirano M Nonaka I. ETFDH mutations, CoQ10 levels, and respiratory chain activities in patients with riboflavin-responsive multiple acyl-CoA dehydrogenase deficiency. Neuromuscul Disord. (2009) 19:212–6. 10.1016/j.nmd.2009.01.008 19249206 PMC10409523

[11] Ohkuma A Noguchi S Sugie H Malicdan MC Fukuda T Shimazu K. Clinical and genetic analysis of lipid storage myopathies. Muscle Nerve. (2009) 39:333–42. 10.1002/mus.21167 19208393 PMC10444629

[12] Wen B Dai T Li W Zhao Y Liu S Zhang C. Riboflavin-responsive lipid-storage myopathy caused by ETFDH gene mutations. J Neurol Neurosurg Psychiatry. (2010) 81:231–6. 10.1136/jnnp.2009.176404 19758981

[13] Lan MY Fu MH Liu YF Huang CC Chang YY Liu JS. High frequency of ETFDH c.250G>A mutation in Taiwanese patients with late-onset lipid storage myopathy. Clin Genet. (2010) 78:565–9. 10.1111/j.1399-0004.2010.01421.x 20370797

[14] Tojo M Gunji T Yamaguchi S Shimizu N Koga Y Nonaka I. A case of riboflavin-responsive multiple acyl-CoA dehydrogenase deficiency (glutaric aciduria type II). No To Hattatsu. (2000) 32:163–8. 10.11251/ojjscn1969.32.163 10723193

[15] Wolfe LA He M Vockley J Payne N Rhead W Hoppel C. Novel ETF dehydrogenase mutations in a patient with mild glutaric aciduria type II and complex II-III deficiency in liver and muscle. J Inherit Metab Dis. (2010) 33(Suppl 3):S481–7. 10.1007/s10545-010-9246-8 21088898 PMC3970109

[16] Xi D Lu J Zhao C Lin J Luo S Zhu W. Clinical features and electron transfer flavoprotein dehydrogenase gene mutation analysis in 35 Chinese patients with lipid storage myopathy. Chin J Neurol. (2011) 44:314–21. 10.3760/cma.j.issn.1006-7876.2011.05.006

[17] Izumi R Suzuki N Nagata M Hasegawa T Abe Y Saito Y. A case of late onset riboflavin-responsive multiple acyl-CoA dehydrogenase deficiency manifesting as recurrent rhabdomyolysis and acute renal failure. Intern Med. (2011) 50:2663–8. 10.2169/internalmedicine.50.5172 22041377

[18] Lämmer AB Rolinski B Ahting U Heuss D. Multiple acyl-CoA-dehydrogenase deficiency (MADD)–a novel mutation of electron-transferring-flavoprotein dehydrogenase ETFDH. J Neurol Sci. (2011) 307:166–7. 10.1016/j.jns.2011.05.001 21616504

[19] Scheicht D Werthmann ML Zeglam S Holtmeier J Holtmeier W Strunk J. Muscle weakness and early stages of liver failure in a 22-year-old man. Internist (Berl). (2013) 54:1016–22. 10.1007/s00108-013-3329-1 23900454

[20] Fitzgerald M Crushell E Hickey C. Cyclic vomiting syndrome masking a fatal metabolic disease. Eur J Pediatr. (2013) 172:707–10. 10.1007/s00431-012-1852-z 23052622

[21] Xi J Wen B Lin J Zhu W Luo S Zhao C. Clinical features and ETFDH mutation spectrum in a cohort of 90 Chinese patients with late-onset multiple acyl-CoA dehydrogenase deficiency. J Inherit Metab Dis. (2014) 37:399–404. 10.1007/s10545-013-9671-6 24357026

[22] Rosenbohm A Süssmuth SD Kassubek J Müller HP Pontes C Abicht A. Novel ETFDH mutation and imaging findings in an adult with glutaric aciduria type II. Muscle Nerve. (2014) 49:446–50. 10.1002/mus.23979 23893693

[23] Zhuo Z Jin P Li F Li H Chen X Wang H. A case of late-onset riboflavin responsive multiple acyl-CoA dehydrogenase deficiency (MADD) with a novel mutation in ETFDH gene. J Neurol Sci. (2015) 353:84–6. 10.1016/j.jns.2015.04.011 25913573

[24] Ersoy EO Rama D Ünal Ö Sivri S Topeli A. Glutaric aciduria type 2 presenting with acute respiratory failure in an adult. Respir Med Case Rep. (2015) 11;15:92–4. 10.1016/j.rmcr.2015.02.009 26236614 PMC4501457

[25] Fu HX Liu XY Wang ZQ Jin M Wang DN He JJ. Significant clinical heterogeneity with similar ETFDH genotype in three Chinese patients with late-onset multiple acyl-CoA dehydrogenase deficiency. Neurol Sci. (2016) 37:1099–05. 10.1007/s10072-016-2549-2 27000805

[26] Dai D Wen F Zhou S Chen S. Clinical features and gene mutations in a patient with multiple aeyl-CoA dehydrogenase deficiency with severe fatty liver. Zhonghua Yi Xue Yi Chuan Xue Za Zhi. (2016) 33:191–4. 10.3760/cma.j.issn.1003-9406.2016.02.014 27060313

[27] Yamada K Kobayashi H Bo R Takahashi T Purevsuren J Hasegawa Y. Clinical, biochemical and molecular investigation of adult-onset glutaric acidemia type II: Characteristics in comparison with pediatric cases. Brain Dev. (2016) 38:293–301. 10.1016/j.braindev.2015.08.011 26403312

[28] Béhin A Acquaviva-Bourdain C Souvannanorath S Streichenberger N Attarian S Bassez G. Multiple acyl-CoA dehydrogenase deficiency (MADD) as a cause of late-onset treatable metabolic disease. Rev Neurol (Paris). (2016) 172:231–41. 10.1016/j.neurol.2015.11.008 27038534

[29] Cui YJ Song CL Cheng YB. Paroxysmal muscle weakness, liver enlargement, and hypoglycemia in a boy. Zhongguo Dang Dai Er Ke Za Zhi. (2017) 19:1104–08. 10.7499/j.issn.1008-8830.2017.10.014 29046209 PMC7389283

[30] Angelini C Tavian D Missaglia S. Heterogeneous phenotypes in lipid storage myopathy due to ETFDH gene mutations. JIMD Rep. (2018) 38:33–40. 10.1007/8904_2017_27 28456887 PMC5874208

[31] Van der Westhuizen FH Smuts I Honey E Louw R Schoonen M Jonck LM. A novel mutation in ETFDH manifesting as severe neonatal-onset multiple acyl-CoA dehydrogenase deficiency. J Neurol Sci. (2018) 15;384:121–5. 10.1016/j.jns.2017.11.012 29249369

[32] Fan X Xie B Zou J Luo J Qin Z D'Gama AM. Novel ETFDH mutations in four cases of riboflavin responsive multiple acyl-CoA dehydrogenase deficiency. Mol Genet Metab Rep. (2018) 16:15–9. 10.1016/j.ymgmr.2018.05.007 29988809 PMC6031868

[33] Chen W Zhang Y Ni Y Cai S Zheng X Mastaglia FL. Late-onset riboflavin-responsive multiple acyl-CoA dehydrogenase deficiency (MADD): case reports and epidemiology of ETFDH gene mutations. BMC Neurol. (2019) 19:330. 10.1186/s12883-019-1562-5 31852447 PMC6921586

[34] Hu G Zeng J Wang C Zhou W Jia Z Yang J. A Synonymous Variant c.579A>G in the ETFDH gene caused exon skipping in a patient with late-onset multiple acyl-coa dehydrogenase deficiency: a case report. Front Pediatr. (2020) 8:118. 10.3389/fped.2020.00118 32292771 PMC7119189

[35] Soldath P Lund A Vissing J. Late-onset MADD: a rare cause of cirrhosis and acute liver failure? Acta Myol. (2020) 39:19–23. 10.36185/2532-1900-003 32607475 PMC7315895

[36] Santananukarn M Amornvit J Pasutharnchat N Jongpiputvanich S. Needle EMG, a Jigsaw to disclose lipid storage myopathy due to multiple Acyl-CoA dehydrogenase deficiency. Am J Phys Med Rehabil. (2020) 99:e71–e4. 10.1097/PHM.0000000000001230 31136308

[37] Pan XQ Chang XL Zhang W Meng HX Zhang J Shi JY. Late-onset multiple acyl-CoA dehydrogenase deficiency with cardiac syncope: A case report. World J Clin Cases. (2020) 8:995–1001. 10.12998/wjcc.v8.i5.995 32190638 PMC7062611

[38] Olsen RK Andresen BS Christensen E Bross P Skovby F Gregersen N. Clear relationship between ETF/ETFDH genotype and phenotype in patients with multiple acyl-CoA dehydrogenation deficiency. Hum Mutat. (2003) 22:12–23. 10.1002/humu.10226 12815589

[39] Xiao C Astiazaran-Symonds E Basu S Kisling M Scaglia F Chapman KA. Mitochondrial energetic impairment in a patient with late-onset glutaric acidemia Type 2. Am J Med Genet A. (2020) 182:2426–31. 10.1002/ajmg.a.61786 32804429 PMC8543298

[40] Nilipour Y Fatehi F Sanatinia S Bradshaw A Duff J Lochmüller H. Multiple acyl-coenzyme A dehydrogenase deficiency shows a possible founder effect and is the most frequent cause of lipid storage myopathy in Iran. J Neurol Sci. (2020) 411:116707. 10.1016/j.jns.2020.116707 32007756

[41] Lian L Chen D Li J Tan S Que J Feng H. Late-onset MADD in Yemen caused by a novel ETFDH mutation misdiagnosed as ADEM. Mult Scler Relat Disord. (2020) 13;48:102689. 10.1016/j.msard.2020.102689 33383363

[42] Alfani R Vassallo E De Anseris AG Nazzaro L D'Acunzo I Porfito C. Pediatric fatty liver and obesity: not always just a matter of non-alcoholic fatty liver disease. Children (Basel). (2018) 5:169. 10.3390/children5120169 30551665 PMC6306738

[43] Vajro P Maddaluno S Veropalumbo C. Persistent hypertransaminasemia in asymptomatic children: a stepwise approach. World J Gastroenterol. (2013) 19:2740–51. 10.3748/wjg.v19.i18.2740 23687411 PMC3653148

[44] Vajro P Lenta S Socha P Dhawan A McKiernan P Baumann U. Diagnosis of nonalcoholic fatty liver disease in children and adolescents: position paper of the ESPGHAN Hepatology Committee. J Pediatr Gastroenterol Nutr. (2012) 54:700–13. 10.1097/MPG.0b013e318252a13f 22395188

[45] Hegarty R Singh S Bansal S Fitzpatrick E Dhawan A. NAFLD to MAFLD in adults but the saga continues in children: an opportunity to advocate change: Letter regarding “A new definition for metabolic dysfunction-associated fatty liver disease: an international expert consensus statement”. J Hepatol. (2021) 74:991–2. 10.1016/j.jhep.2020.12.032 33493527

[46] Richards S Aziz N Bale S Bick D Das S Gastier-Foster J. ACMG Laboratory Quality Assurance Committee. Standards and guidelines for the interpretation of sequence variants: a joint consensus recommendation of the American College of Medical Genetics and Genomics and the Association for Molecular Pathology. Genet Med. (2015) 17:405–24. 10.1038/gim.2015.30 25741868 PMC4544753

[47] Goh LL Lee Y Tan ES Lim JSC Lim CW Dalan R. Patient with multiple acyl-CoA dehydrogenase deficiency disease and ETFDH mutations benefits from riboflavin therapy: a case report. BMC Med Genomics. (2018) 11:37. 10.1186/s12920-018-0356-8 29615056 PMC5883299

[48] Cornelius C. Byron I. Hargreaves P.F. Guerra A.K. Furdek J. Land. Secondary coenzyme Q10 deficiency and oxidative stress in cultured fibroblasts from patients with riboflavin responsive multiple Acyl-CoA dehydrogenation deficiency. Hum. Mol. Genet. (2013) 22:3819–27 10.1093/hmg/ddt232 23727839

