# Supplementary figures and images for: Hepatic Presentation of Late-Onset Multiple Acyl-CoA Dehydrogenase Deficiency (MADD): Case Report and Systematic Review
Source: Front Pediatr. 2021 May 10;9:672004. doi: 10.3389/fped.2021.672004 (PMC8143529; doi:10.3389/fped.2021.672004)

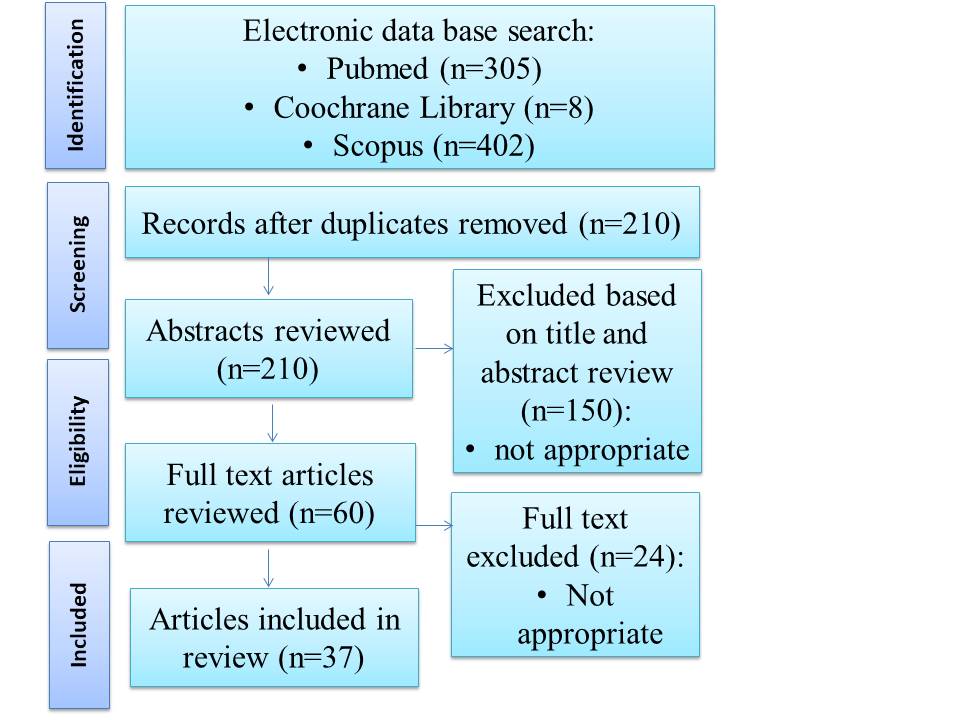

Supplement: Supplementary Figure 1 — Flowchart of literature search results. Preferred Reporting Items for Systematic Reviews and Meta-Analyses (PRISMA) flowchart of literature search results. From: Moher D, Liberati A, Tetzlaff J, Altman DG, The PRISMA Group (2009). Preferred Reporting Items for Systematic Reviews and Meta-Analyses: The PRISMA Statement. PLoS Med 6(7):e1000097. [file Image_1.JPEG]
