# Supplementary material for: Hepatic Presentation of Late-Onset Multiple Acyl-CoA Dehydrogenase Deficiency (MADD): Case Report and Systematic Review
Source: Front Pediatr. 2021 May 10;9:672004. doi: 10.3389/fped.2021.672004 (PMC8143529; doi:10.3389/fped.2021.672004)
Supplement: Supplementary Table 1 — Serum acylcarnitines, aminoacids, and urinary organic acids at presentation and during the follow-up. [file Table_1.DOCX]

| **Table S1. S**erum acylcarnitines, aminoacids, and urinary oganic acids. Shaded areas indicate abnormal values | | | | | | | | | | | | | | | | | |
| --- | --- | --- | --- | --- | --- | --- | --- | --- | --- | --- | --- | --- | --- | --- | --- | --- | --- |
| **Plasma C3 to C16 acylcarnitine concentrations ( µmol/l)** | | | | | | | | | | | | | | | | | |
|  | **July 2019** | | **September 2019** | | **October 2019** | | **November 2019** | | **Dicember 2019** | | **January**  **2020** | | **February 2020** | | **August**  **2020** | | **Reference**  **values (µmol/l)** |
| C4 | 1.10 ↑ | | 5.91↑ | | 2.71↑ | | 2.74↑ | | 3.48 ↑ | | 4.68 ↑ | | 0.99 ↑ | | 1.72 ↑ | | 0.12-0.42 |
| C5 | 0.47 ↑ | | 0.48↑ | | 0.92↑ | | 0.69↑ | | 0.42 ↑ | | 0.56 ↑ | | 0.17 | | 0.47↑ | | 0.05-0.24 |
| C6 | - | | 1.37↑ | | 0.40↑ | | 0.28↑ | | 1.46 ↑ | | 1.88 ↑ | | 0.31↑ | | 0.62↑ | | 0.04-0.18 |
| C8 | 0.40 ↑ | | 2.58↑ | | 0.89↑ | | 0.71↑ | | 3.47 ↑ | | 5.91 ↑ | | 2.29 ↑ | | 0.22 | | 0.07-0.25 |
| C10 | 0.82 ↑ | | 3.37↑ | | 1.71↑ | | 1.26↑ | | 4.14 ↑ | | 7.18 ↑ | | 3.56 ↑ | | 0.16 | | 0.09-0.43 |
| C12:1 | 0.50 ↑ | | 0.43↑ | | 0.36↑ | | 0.44↑ | | 0.29 ↑ | | 0.40 ↑ | | 0.23 ↑ | | 0.24↑ | | 0.04-0.20 |
| C12 | 0.89 ↑ | | 0.86↑ | | 1.38↑ | | 1.16↑ | | 0.61 ↑ | | 1.24 ↑ | | 0.48 ↑ | | 0.34↑ | | 0.04-0.21 |
| C14:2 | 0.41 ↑ | | 0.29↑ | | 0.19↑ | | 0.22↑ | | 0.17↑ | | 0.30 ↑ | | 0.12 | | 0.17 | | 0.02-0.16 |
| C14:1 | 1.86 ↑ | | 0.83↑ | | 1.34↑ | | 1.66↑ | | 0.75 ↑ | | 0.92 ↑ | | 0.40 ↑ | | 0.43↑ | | 0.02-0.20 |
| C14 | 1.33 ↑ | | 0.42↑ | | 0.87↑ | | 1.03↑ | | 0.23 ↑ | | 0.42 ↑ | | 0.24 ↑ | | 0.27↑ | | 0.03-0.15 |
| C16:1 | 2.49 ↑ | | 0.07 | | 1.13↑ | | 1.21↑ | | 0.42 ↑ | | 0.59 ↑ | | 0.21 ↑ | | 0.33↑ | | 0.01-0.07 |
| C16 | 2.05 ↑ | | 0.68↑ | | 1.05↑ | | 1.37↑ | | 0.31 ↑ | | 0.62 ↑ | | 0.26 ↑ | | 0.36↑ | | 0.01-0.23 |
| C16 OH | 0.08 ↑ | | 0.08↑ | | 0.05↑ | | 0.10↑ | | 0.03 | | 0.02 | | 0.02 | | 0.03 | | 0.01-0.05 |
| C18:1 | 1.58 ↑ | | 0.43↑ | | 0.63↑ | | 0.92↑ | | 0.29 | | 0.31 | | 0.19 | | 0.34 | | 0.02-0.34 |
| C18 | 0.53 ↑ | | 0.26↑ | | 0.37↑ | | 0.44↑ | | 0.08 | | 0.13 | | 0.11 | | 0.11 | | 0.01-0.18 |
| C18:1OH | 0.12 ↑ | | 0.05↑ | | 0.04↑ | | 0.08↑ | | 0.03 | | 0.01 | | 0.02 | | 0.04 | | 0.01-0.07 |
|  | | | | | | | | | | | | | | | | | |
| **Serum Aminoacids ( µmol/l)** | | | | | | | | | | | | | | | | | |
| Asparagine | 168 ↑ | | **(-)** | | 258↑ | | **NOT DONE** | | 65 | | 73 | | 67 | | **NOT DONE** | | 43-87 |
| Glutamine | 305 ↓ | | **(-)** | | 520 | |  | | 599 | | 671 | | 776 | |  | | 432-871 |
| Histidine | 135 ↑ | | **(-)** | | 117 | |  | | 99 | | 117 | | 110 | |  | | 72-131 |
| Sarcosine | 242 ↑ | | **(-)** | | **(-)** | |  | | **(-)** | | **(-)** | | **(-)** | |  | | 0-50 |
| Proline | 1629 ↑ | | **(-)** | | **(-)** | |  | | 339 | | 363 ↑ | | 436↑ | |  | | 50-350 |
| Lysine | - | | **(-)** | | 209 | |  | | (-) | | 126 ↓ | | 130↓ | |  | | 150-286 |
|  | | | | | | | | | | | | | | | | | |
| **Urinary organic acids** (mmol/mol of creat.) | | | | | | | | | | | | | | | | | |
| Lactic acid | | **(-)** | | **(-)** | | **(-)** | | **NOT DONE** | | 17 | | 18 | | 20 | 34 | 1-25 | |
| Isobutyric 2OH a. | | **(-)** | | **(-)** | | **(-)** | |  | | 5 | | 10 ↑ | | 2 | - | 0-2 | |
| Glycolic a. | | 9 ↓ | | 7↓ | | 10 | |  | | 9 | | 13 ↓ | | 14 ↓ | 61 | 43-172 | |
| 3-OH butyric a. | | 10 ↑ | | 7 | | 10 | |  | | (-) | | 2 | | 7 ↓ | 8 | n.d.- 7.6 | |
| Ethylmalonic a. | | 141 ↑ | | 68 ↑ | | 159 ↑ | |  | | 72 ↑ | | 101 ↑ | | 17 ↑ | 161↑ | n.d.-8.4 | |
| Methylsuccinic a. | | 40 ↑ | | 15 ↑ | | 34 ↑ | |  | | 19 ↑ | | 34 ↑ | | 5 ↑ | 29↑ | n.d.- 4.4 | |
| Fumaric a. | | 4 ↑ | | 1 | | 3 | |  | | - | | 3 | | 1 | 4 | n.d.-3.7 | |
| Glutaric a. | | 92 ↑ | | 4 | | 40 ↑ | |  | | 6 ↑ | | 9 ↑ | | 6 ↑ | 17↑ | n.d.-3.8 | |
| Isovalerilglicine | | 40 ↑ | | **(-)** | | 23 | |  | | 5 | | 9 ↑ | | 7 ↑ | 3 | n.d. | |
| Adipic ac. | | 787 ↑ | | 9 | | 338 | |  | | **(-)** | | 15 ↑ | | **(-)** | 84 | n.d.-5.3 | |
| Pyruvic acid | | 30 ↑ | | 27 ↑ | | 30 ↑ | |  | | 35 ↑ | | 25 ↑ | | 28 ↑ | 40↑ | 3.5-17.3 | |
| 2-OH glutaric a. | | 20 ↑ | | 3 | | 39 ↑ | |  | | 4 | | 6 | | **(-)** | 25↑ | 1.3-13.9 | |
| Pimelic a. | | 24 ↑ | | 3 | | 15 ↑ | |  | | 5 | | **(-)** | | 1 | 10↑ | 0-2 | |
| Suberic a. | | 365 ↑ | | **(-)** | | 62 | |  | | **(-)** | | **(-)** | | **(-)** | 19↑ | n.d.-8.8 | |
| Cis-aconitic a. | | 11 ↓ | | 7 ↓ | | 19 | |  | | 18 ↓ | | **(-)** | | 9 ↓ | 26 | 20.5-135 | |
| Hippuric a. | | 28 ↓ | | 83 ↓ | | 75 | |  | | 231 | | 79 ↓ | | 60 ↓ | 56 | 119-1390 | |
| Sebacic a. | | 69 ↑ | | **(-)** | | 3 | |  | | **(-)** | | 2 ↑ | | **(-)** | 11↑ | n.d-1.4 | |
| Palmitic a. | | 5 ↓ | | 10 | | 20 | |  | | 15 | | 38 ↑ | | **(-)** | 16 | 6-23 | |
| Stearic a. | | 17↑ | | 20 ↑ | | 36 | |  | | 18 ↑ | | 53 ↑ | | 50 ↑ | 16↑ | 1.6-6.6 | |
| Glyoxylic a. | | 13 ↑ | | 20 ↑ | | 12 ↑ | |  | | 12 ↑ | | 6 ↑ | | 17 ↑ | 13↑ | 0.2-5.7 | |
| α ketoglutaric a. | | - | | 24 ↓ | | 30 | |  | | 24 ↓ | | 22 ↓ | | 17 ↓ | 40 | 29.8-117 | |
| **Symbols and abbreviations:** **↑**: increased**. ↓:** decreased. **a**: acid. **(-):** not reported. **n.d.:** not determinated | | | | | | | | | | | | | | | | | |
