# Supplementary material for: Hepatic Presentation of Late-Onset Multiple Acyl-CoA Dehydrogenase Deficiency (MADD): Case Report and Systematic Review
Source: Front Pediatr. 2021 May 10;9:672004. doi: 10.3389/fped.2021.672004 (PMC8143529; doi:10.3389/fped.2021.672004)
Supplement: Supplementary Table 2 — Key words used for PUBMED, Scopus and Cochrane library search. [file Table_2.DOCX]

| **Table S2.** Key words used for PUBMED, SCOPUS and Cochrane library search |
| --- |
| MADD and fatty liver; Multiple acyl-CoA dehydrogenase deficiency and fatty liver; Glutaric aciduria, type 2 and fatty liver; MADD and Liver disease; Multiple acyl-CoA dehydrogenase deficiency and liver disease; Glutaric aciduria type 2 and liver disease; MADD and hypertransaminasemia; Glutaric aciduria type 2 and hypertransaminasemia; multiple deficit of acyl-CoA dehydrogenase and hypertransaminasemia; MADD and steatosis; multiple deficit of acyl-CoA dehydrogenase and steatosis; Glutaric aciduria type 2 and steatosis.  ETF mutation and fatty liver; ETF mutation and liver disease; ETF mutation and steatosis; ETF mutation and hypertransaminasemia; MADD and ETFDH; ETFDH. |
