# Supplementary material for: Hepatic Presentation of Late-Onset Multiple Acyl-CoA Dehydrogenase Deficiency (MADD): Case Report and Systematic Review
Source: Front Pediatr. 2021 May 10;9:672004. doi: 10.3389/fped.2021.672004 (PMC8143529; doi:10.3389/fped.2021.672004)
Supplement: Supplementary file 4 [file Table_3.DOCX]

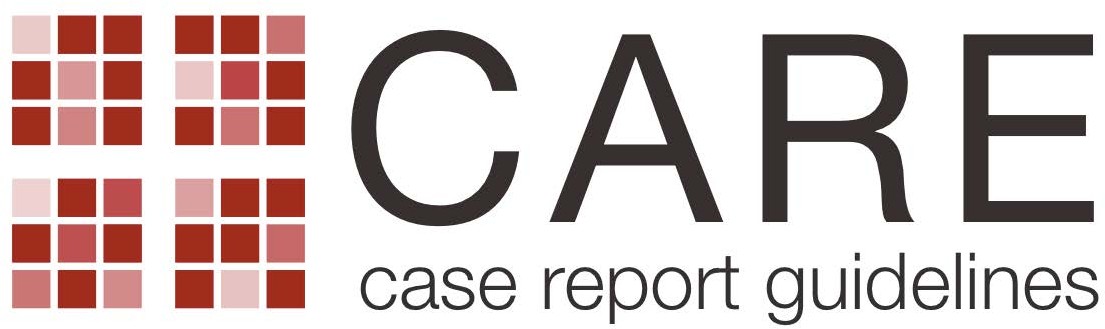
CARE Checklist of information to include when writing a case report
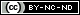


**Topic Item Checklist item description Reported on Line**

**Title 1** The diagnosis or intervention of primary focus followed by the words “case report” 2

**Key Words 2** 2 to 5 key words that identify diagnoses or interventions in this case report, including "case report" 34

**Abstract**

**(no references)**

**3a** Introduction: What is unique about this case and what does it add to the scientific literature? 42

**3b** Main symptoms and/or important clinical findings 44-45

**3c** The main diagnoses, therapeutic interventions, and outcomes 56-58

**3d** Conclusion—What is the main “take-away” lesson(s) from this case? 62-63

**Introduction 4** One or two paragraphs summarizing why this case is unique (**may include references**) 91-92

**Patient Information 5a** De-identified patient specific information 110

**5b** Primary concerns and symptoms of the patient 110-111

**5c** Medical, family, and psycho-social history including relevant genetic information 110-116

**5d** Relevant past interventions with outcomes 117-123

**Clinical Findings**

**Timeline**

**Diagnostic Assessment**

**Therapeutic Intervention**

**Follow-up and Outcomes**

1. Describe significant physical examination (PE) and important clinical findings 124-133
2. Historical and current information from this episode of care organized as a timeline 124-133

**8a** Diagnostic testing (such as PE, laboratory testing, imaging, surveys). 134-160

**8b** Diagnostic challenges (such as access to testing, financial, or cultural) 134-137

**8c** Diagnosis (including other diagnoses considered) 157-158

**8d** Prognosis (such as staging in oncology) where applicable /

**9a** Types of therapeutic intervention (such as pharmacologic, surgical, preventive, self-care) 161

**9b** Administration of therapeutic intervention (such as dosage, strength, duration) 161

**9c** Changes in therapeutic intervention (with rationale) /

**10a** Clinician and patient-assessed outcomes (if available) 161-167

**10b** Important follow-up diagnostic and other test results 163-167

**10c** Intervention adherence and tolerability (How was this assessed?) /

**10d** Adverse and unanticipated events /

**Discussion 11a** A scientific discussion of the strengths AND limitations associated with this case report 208-229

**11b** Discussion of the relevant medical literature **with references** 170-187

**11c** The scientific rationale for any conclusions (including assessment of possible causes) 232-233

**11d** The primary “take-away” lessons of this case report (without references) in a one paragraph conclusion 234-238

**Patient Perspective 12** The patient should share their perspective in one to two paragraphs on the treatment(s) they received /

**Informed Consent 13** Did the patient give informed consent? Please provide if requested . . . . . . . . . . . . . . . . . . . . . . . . . . . . . . . . . . . . . . **Yes X No**
